# Risk of Glaucoma in Patients Receiving Hemodialysis and Peritoneal Dialysis: A Nationwide Population-Based Cohort Study

**DOI:** 10.3390/ijerph17186774

**Published:** 2020-09-17

**Authors:** Chen-Chee Lim, Chia-Yi Lee, Fu-Chin Huang, Jing-Yang Huang, Jia-Horung Hung, Shun-Fa Yang

**Affiliations:** 1 Department of Ophthalmology, National Cheng Kung University Hospital, College of Medicine, National Cheng Kung University, Tainan 704, Taiwan; internj91@gmail.com (C.-C.L.); huangfc@mail.ncku.edu.tw (F.-C.H.); 2 Department of Ophthalmology, Show Chwan Memorial Hospital, Changhua 500, Taiwan; ao6u.3msn@hotmail.com; 3 Institute of Clinical Medicine, College of Medicine, National Cheng Kung University, Tainan 704, Taiwan; wchinyang@gmail.com; 4 Department of Medical Research, Chung Shan Medical University Hospital, Taichung 402, Taiwan; 5 Institute of Medicine, Chung Shan Medical University, Taichung 402, Taiwan

**Keywords:** end-stage renal disease, hemodialysis, intraocular pressure

## Abstract

This paper investigated the incidence and risk of newly diagnosed glaucoma after the initiation of maintenance dialysis in Taiwan. A case–control study was conducted using the National Health Insurance Research Database (NHIRD) in Taiwan. There were 3949 patients with dialysis in the study group and 78,980 non-dialysis subjects matched by age and sex in the comparison group. The incidence of newly diagnosed glaucoma after the initiation of maintenance dialysis was analyzed based on the diagnostic code for glaucoma. Patients with dialysis had a higher risk of glaucoma (adjusted hazard ratio (aHR): 1.270; 95% confidence interval (CI): 1.035–1.560) than patients without dialysis. The incidence rate of glaucoma was 8.18 per 10,000 person months in the dialysis group, which was higher than that in the non-dialysis group (5.01 per 10,000 person months). Patients with dialysis exhibited a significantly higher risk of angle-closure glaucoma (ACG) (aHR: 1.550; 95% CI: 1.074–2.239). In contrast, there was no significant risk of developing open-angle glaucoma or normal-tension glaucoma in dialysis patients. Our data suggest that dialysis patients are more susceptible to ACG. Regular ophthalmic examinations may be useful in patients with dialysis to identify high-risk individuals with glaucoma, and preventive measures can be applied to avoid permanent vision loss as soon as intraocular pressure (IOP) elevation is identified.

## 1. Introduction

End-stage renal disease (ESRD) is a chronic condition in which patients rely on either maintenance dialysis or a renal transplant, and it is also associated with a significant socioeconomic burden. Taiwan has been reported to have the highest incidence (493 per million) and prevalence (3392 per million) of treated ESRD in the world [1]; 87.5% of these patients receive hemodialysis (HD), while 8.5% receive peritoneal dialysis (PD) [2,3]. In 2010, it was estimated that 2.6 million individuals worldwide received renal replacement therapy, and the number is estimated to be more than 5.4 million by 2030 [4].

Several studies have discussed intraocular pressure (IOP) changes during HD since the first demonstration of an increase in IOP during dialysis in 1964 [5,6]. Some studies have revealed that IOP may be elevated [6,7,8,9,10], some have shown that IOP does not change [11,12], and some have demonstrated that IOP decreases [13,14,15,16,17]. However, these previous results were either limited by small patient numbers, a short follow-up period, or lack of a comparative group. Moreover, the relationship between IOP changes and the subsequent development of glaucoma is also not well studied. Doshiro et al. [14] showed that IOP decreased during HD possibly due to plasma colloid osmotic pressure increase; however, patients with HD for more than 12 years had a tendency for increased IOP.

It has become an accepted concept that eyes with impaired aqueous outflow facilities, such as shallow anterior chambers of angle closure, may have a significant increase in IOP during HD [12]. Because of the high prevalence of occludable angles in Taiwanese people [18], it is of clinical importance to investigate the development of glaucoma after the initiation of maintenance dialysis in Taiwan. Utilizing a nationwide population-based dataset in Taiwan, namely, the National Health Insurance Research Database (NHIRD), we designed a cohort study to investigate the incidence and risk of newly diagnosed glaucoma after the initiation of maintenance dialysis in Taiwan. 

## 2. Materials and Methods 

### 2.1. Data Source

This retrospective population-based cohort study was approved by the National Health Insurance Administration and the Institutional Review Board of Chung Shan Medical University (Registration Number: CSMUH CS2-15061). The claim data originated from the National Health Insurance Research Database (NHIRD) in Taiwan. National Health Insurance (NHI) in Taiwan is a nationwide healthcare program that was launched in 1995. NHI covers 99.82% of Taiwan residents, with a total of 23.948 million by the end of 2018 [19]. The NHIRD provides individuals’ encrypted information—date of birth, sex, place of residence, inpatient and outpatient services, details of medications, intervention procedures, date of admission and discharge, and diagnosis records (based on the International Classification of Diseases, 9th Revision, Clinical Modification (ICD-9-CM)). We utilized the Longitudinal Health Insurance Database (LHID), a dataset released by the NHIRD, which contains all of the original claim data (from 1997 to 2013) of 1 million individuals who were randomly sampled from the NHIRD. Several high-quality studies have shown the accuracy of the NHIRD [20,21,22,23]. 

### 2.2. Patient Selection

**Figure 1:**
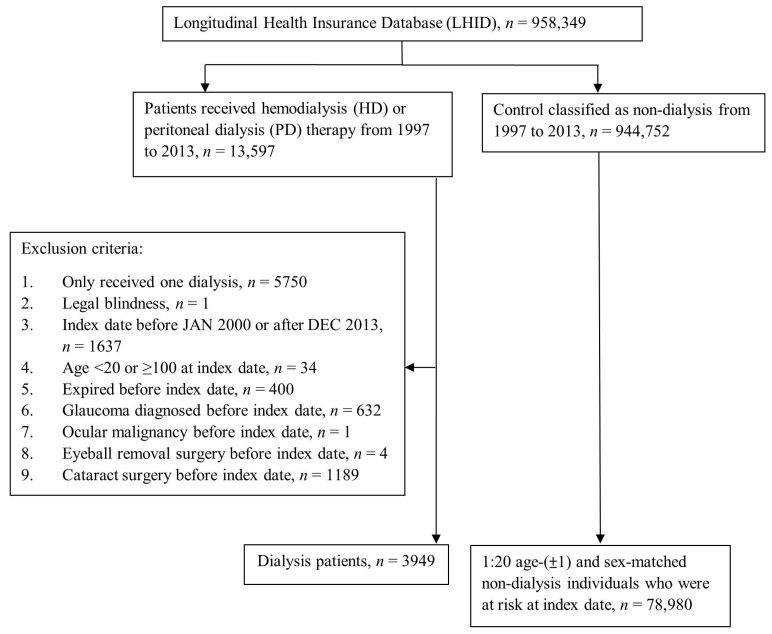
Flow diagram showing the selection of the study participants with and without dialysis. Index date: For dialysis patients, the index date was 90 days after the first dialysis. For non-dialysis individuals, the index date nested with the paired dialysis patients. All study participants were at risk on the index date.

Our study cohort included individuals newly diagnosed with ESRD who had received HD or PD more than twice, and whose treatment period of HD or PD was more than three months during the study period from January 1997 to December 2013. The index date was defined as 90 days after the first dialysis. To evaluate the correlation between dialysis and new-onset glaucoma, the following exclusion criteria were defined: (1) Diagnosis of glaucoma before the index date; (2) legal blindness at any time; (3) an index date earlier than 2000; (4) age younger than 20 or older than 100; (5) deceased before the index date; (6) diagnosis of an ocular tumor before the index date; (7) absence of an eyeball or anophthalmos before the index date; (8) cataract surgery before the index date. After the exclusions, 3949 patients with dialysis remained in the study cohort. This study design compared the difference in the risk of glaucoma between dialysis and non-dialysis cohorts. Each dialysis patient was assigned to 20 controls matched by age and sex, and a total of 78,980 comparison cases were included in this study (Figure 1).

### 2.3. Main Outcome Measurement

The primary outcome was defined as newly diagnosed glaucoma after the initiation of maintenance dialysis based on the diagnostic code for glaucoma (ICD-9 codes: 365.1x, 365.2x, 365.7x, and 365.9; ICD-10 codes: H40.1x, H40.2x, H40.89, and H40.9) after the index date. The study period was from 1 January 2000 to 31 December 2013. Subgroups of glaucoma, including open-angle glaucoma (OAG) (ICD-9 code: 365.10, 365.11, 365.13, and 365.15; ICD-10: H40.10x, H40.11x, H40.13x, and H40.15x), angle-closure glaucoma (ACG) (ICD-9: 365.2x; ICD-10: H40.2x), and normal-tension glaucoma (NTG) (ICD-9: 365.12; ICD-10: H40.12x), were analyzed to identify their relationships with dialysis. To ensure accuracy, only patients with diagnostic codes of glaucoma made by ophthalmologists (department code: 10), ≥2 clinic visits, and prescribed with glaucoma medications (categorized using the Anatomical Therapeutic Chemical (ATC) drug code, including sympathomimetics in glaucoma therapy (S01EA), parasympathomimetics (S01EB), carbonic anhydrase inhibitors (S01EC), beta blocking agents (S01ED), and prostaglandin analogues (S01EE)) were included in this study. Moreover, glaucoma-related codes that indicate identifiable factors (e.g., pigment dispersion in pigmentary glaucoma, pseudoexfoliative material of pseudoexfoliation syndrome, and steroid-induced glaucoma), suspected glaucoma, ocular hypertension, steroid responders, anatomical narrow angle, and pre-glaucoma were excluded from the current study to avoid overestimation and confusion of the diagnosis.

### 2.4. Identification of Comorbidities

We identified the comorbidities of each participant to evaluate their health status and to investigate the correlation between glaucoma and comorbidities. Comorbidities included hypertension, diabetes mellitus, hyperlipidemia, ischemic heart diseases, congestive heart failure, cerebrovascular disease, dementia, liver disease, hemiplegia or paraplegia, uveitis, retinal vessel occlusion, non-proliferative diabetic retinopathy, and proliferative diabetic retinopathy. Participants were confirmed to have these comorbidities if the relevant ICD codes were diagnosed once or more at the inpatient service or twice or more at the outpatient service with a minimal interval of more than 30 days within one year before enrollment.

### 2.5. Statistical Analysis 

All analyses were conducted using SAS version 9.4 (SAS Institute Inc., Cary, NC, USA). After 1:20 matching, the absolute standardized difference (ASD) was used to evaluate the balance between the study and control groups. Student’s *t*-tests were used to compare continuous variables, while chi-square tests or Fisher’s exact tests were used to compare the differences between categorical variables. Calculations of the incidence rate and the corresponding 95% confidence intervals were conducted using the Poisson assumption. Cumulative incidence rates were calculated according to analysis of the cumulative incidence of competing risks. Multiple Cox proportional hazards regressions were used to generate an adjusted hazard ratio (aHR) by integrating individuals’ demographic information and comorbidities. The aHRs of the dialysis, demographics, and comorbidities were analyzed. Glaucoma was divided into three subgroups: OAG, NTG, and ACG. The incidence rates of each subgroup were computed. A *p*-value < 0.05 was regarded as statistically significant.

## 3. Results

### 3.1. Baseline Characteristics of the Study Cohort

A total of 3949 subjects under maintenance dialysis were included in the study group, and another 78,980 non-dialysis subjects served as the control group. The flow chart of subject selection is shown in Figure 1. Table 1 summarizes the differences in the baseline characteristics between the dialysis and non-dialysis groups. The number of people diagnosed with hypertension, diabetes mellitus, ischemic heart diseases, hyperlipidemia, congestive heart failure, cerebrovascular disease, dementia, non-proliferative diabetic retinopathy, and proliferative diabetic retinopathy was significantly higher in the dialysis group than in the non-dialysis group. The frequency of visits to ophthalmologists of the two groups is shown below.

### 3.2. Comparison of the Incidence Rates and Cumulative Risk of Glaucoma between Dialysis and Non-Dialysis Patients

**Figure 2:**
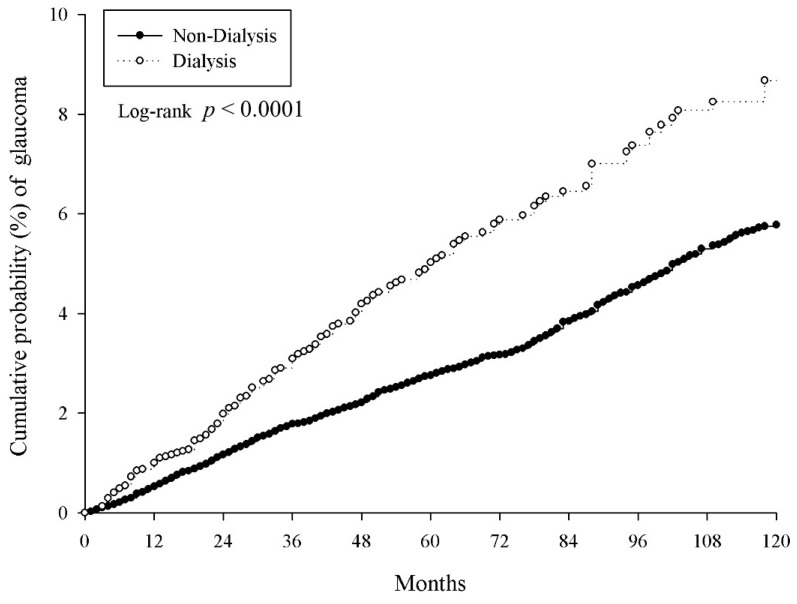
Kaplan–Meier curves of the cumulative probability of glaucoma.

The incidence rates for new glaucoma cases are presented in Table 2. The incidence rate of glaucoma per 10,000 person months was 8.18 (95% CI: 7.01–9.54) in the dialysis group, which was significantly higher than that in the non-dialysis group (5.01; 95% CI: 4.83–5.2). The cumulative incidence of glaucoma in the dialysis group after approximately 120 months of follow-up was significantly greater than that in the non-dialysis group (*p* < 0.0001, log-rank test; Figure 2), and the crude relative risk was 1.618 (95% CI: 1.38–1.897).

### 3.3. Adjusted Hazard Ratios of Glaucoma Related to Dialysis Based on Various Covariates 

The risk of glaucoma development based on the multivariate Cox proportional hazards models is presented in Table 3. After adjustment for potential confounders, the dialysis group was associated with a significantly higher risk of glaucoma (adjusted HR: 1.270; 95% CI: 1.035–1.560). In addition, older age (older than 60 years old), hypertension, diabetes mellitus, ischemic heart disease, hyperlipidemia, uveitis, retinal vessel occlusion, and proliferative diabetic retinopathy were also associated with an increased risk of glaucoma.

### 3.4. Incidence Rate and Adjusted Hazard Ratios of Different Types of Glaucoma and Trabeculectomy Related to Dialysis

After stratifying by different types of glaucoma, subevent analysis demonstrated that the dialysis group had a significantly higher incidence rate of ACG than the control group—2.28 (95% CI: 1.71–3.04) for the dialysis group, and 1.52 (95% CI: 1.43–1.63) for the non-dialysis group (Table 4). After adjusting for potential confounders, dialysis patients were associated with a significantly higher risk of ACG (adjusted HR: 1.550; 95% CI: 1.074–2.239). Furthermore, dialysis was also associated with trabeculectomy, which is a surgical treatment for severe glaucoma (adjusted HR: 3.666; 95% CI: 1.366–9.840). The incidence rate and adjusted HR of OAG and NTG showed no significant differences between the dialysis and non-dialysis groups (Table 4).

## 4. Discussion

To the best of our knowledge, this is the first study to investigate the role of dialysis in the cumulative risk of glaucoma using population-based claims big-data analysis. The large sample size of Taiwan’s NHIRD allowed for a statistically powerful assessment of rare events. In this population-based follow-up study of 3949 patients first receiving HD or PD and 78,980 control cases, the incidence rate of new-onset glaucoma per 10,000 person months was 8.18 (95% CI: 7.01–9.54) in the dialysis group, with a relative risk of 1.618 (95% CI: 1.38–1.897) (Table 2). In subevent analysis, the adjusted hazard ratios for ACG and trabeculectomy for subjects who were undergoing dialysis were 1.550 (95% CI: 1.074–2.239) and 3.666 (95% CI: 1.366–9.840), respectively, compared with the control cases (Table 4). On the other hand, OAG and NTG showed no difference between the dialysis and control groups.

Ocular abnormalities are frequently associated with patients on chronic dialysis, including IOP fluctuation, corneal calcification, retinal hemorrhage, retinal toxicity related to desferrioxamine, anterior ischemic neuropathy, and uremic optic neuropathy [24]. Among them, the relationship between IOP changes and dialysis has been reported with various results [6,7,8,9,10,11,12,13,14,15,16,17,25]. Moreover, there has long been a debate regarding whether transient IOP changes during HD would result in the progression of glaucoma [26]. The present study utilized a population-based claims database, applied multivariate regression methods with adjustment of confounders, and proved that patients receiving dialysis were at a higher risk of developing glaucoma (Table 2 and Table 3).

Our study confirmed the hypothesis that HD has a greater impact on eyes with impaired aqueous outflow facilities, such as a shallow anterior chamber of the ACG. The mechanism of IOP changes during dialysis is still unknown, but the proposed mechanism is as follows: During HD, the dialysis procedure removes osmotically active substances by diffusion, which leads to a decrease in serum osmolality and loss of body fluids. Rapid reduction in plasma osmolality results in disequilibrium between the intraocular fluid and plasma because of the rapid decrease in plasma osmolality and the unchanged ocular osmolality during HD [6]. The relatively high urea concentration in the intraocular fluid compartment during HD [27] might cause fluid shifting from blood plasma to the anterior chamber [12,26,28,29,30]. For eyes with normal outflow facilities, water movement on this scale might not influence the IOP. In contrast, for eyes with impaired aqueous outflow facilities, the increment of IOP is more prominent [12,31]. The decrease in anterior chamber depth during HD further compromises outflow facilities [32,33,34]. Therefore, detailed ophthalmic examination should be conducted among patients receiving dialysis to screen high-risk individuals who have the potential to develop ACG.

Because IOP elevation usually occurs during dialysis, and patients receiving maintenance dialysis have a greater risk of glaucoma, preventive measures during dialysis should be taken with high-risk patients to avoid irreversible vision loss. Conventional HD protocols consist of three sessions per week, and each session lasts approximately three to four hours. For those individuals susceptible to experiencing IOP elevation during HD, chronic elevation in IOP might result in optic nerve damage. Some approaches have been reported to hinder a symptomatic rise in IOP during dialysis, including intravenous mannitol [35], hypertonic sodium dialysis or use of hyperosmotic agents [12], slower urea removal [36], modified dialysis parameters with colloid infusion [30], and intravenous glucose administration [37].

Acute primary angle closure (APAC) is a medical emergency and remains a therapeutic challenge, particularly in ESRD patients. Conventional management of APAC includes medical therapy and laser periphery iridotomy (LPI). However, LPI is extremely difficult to perform due to severe corneal edema during APAC. Patients with APAC frequently present with ocular pain, nausea, and vomiting. Oral medications (including acetazolamide, glycerol, and isosorbide) are often contraindicated in ESRD patients and are unable to be administered when patients are experiencing nausea and vomiting. The iris sphincter may be unresponsive to topical miotic agents at high IOP due to pressure-induced ischemic paralysis of the iris. Rapid IOP reduction is important for relieving ocular symptoms and clearing the cornea for a more definitive procedure, such as LPI or cataract extraction. Intravenous hyperosmotic agents (e.g., mannitol) are the first drugs of choice for APAC. Nevertheless, in ESRD patients, intravenous administration of mannitol should be followed by dialysis to avoid fluid overload [38]. To avoid this challenging conundrum, prophylactic LPI or cataract extraction may be considered a preventive measure in patients with ESRD who are at risk of APAC. Trabeculectomy and Ahmed valve implantation [39] are surgical options for medically uncontrolled IOP. The Xen Gel Stent, an ab interno minimally invasive glaucoma surgery, offers an alternative option for mild to moderate OAG with the advantage of lower ocular surface inflammation compared to topical therapy and trabeculectomy [40].

There are still some limitations to our study. First, identification of the demographic information and associated comorbidities of these patients depended on the accuracy of the ICD-9-CM codes, so coding errors might exist, and the disease codes might be less accurate. Second, the observational cohort study design may not have elucidated causal inference, and the findings of this study should be interpreted with discretion. Third, the prevalence of open-angle glaucoma and normal tension glaucoma is underestimated in the Asian population [41,42] and most of the insurants of Taiwan are Chinese; therefore, it is unclear whether these results can be generalized to other racial/ethnic groups. Fourth, the diagnosis of glaucoma relies only on diagnostic codes made by ophthalmologists without supporting evidence, such as optic disc and visual field analysis, so the results should be interpreted with caution. Fifth, the prevalence of glaucoma in the non-dialysis group may have been underestimated due to limited clinic visits compared to the dialysis group. Sixth, the inclusion and exclusion criteria in our study may have excluded some cases of mild glaucoma under observation without treatment, thus reducing the number of participants; however, this could have enhanced the accuracy of the diagnosis of glaucoma and precluded cases of suspected glaucoma.

## 5. Conclusions

The presented results support the hypothesis that patients with dialysis are at an increased risk of glaucoma compared with non-dialysis individuals. Furthermore, our data suggest that dialysis patients are more susceptible to ACG, which is possibly due to decreased ACD and, subsequently, increased IOP during dialysis. As a consequence, regular ophthalmic examinations may be useful in patients with dialysis to identify high-risk individuals with glaucoma, and preventive measures should be applied to avoid permanent vision loss as soon as IOP elevation is identified.

## Figures and Tables

**Table 1:** Baseline characteristics.

	Non-Dialysis *n* = 78,980	Dialysis *n* = 3949	ASD
Sex			0.000
Female	37,440 (47.40%)	1872 (47.40%)	
Male	41,540 (52.60%)	2077 (52.60%)	
Age			0.003
20–39	5968 (7.56%)	295 (7.47%)	
40–59	30,032 (38.02%)	1504 (38.09%)	
60–79	35,310 (44.71%)	1767 (44.75%)	
80–100	7670 (9.71%)	383 (9.70%)	
Urbanization			0.031
Urban	45,437 (57.53%)	2256 (57.13%)	
Suburban	23,815 (30.15%)	1197 (30.31%)	
Rural	9728 (12.32%)	496 (12.56%)	
Low income	504 (0.64%)	35 (0.89%)	0.029
Length of hospital stay *			3.081
0 days	69,845 (88.43%)	251 (6.36%)	
1–6 days	4371 (5.53%)	365 (9.24%)	
≥7 days	4764 (6.03%)	3333 (84.40%)	
Comorbidity *			
Hypertension	24,337 (30.81%)	3438 (87.06%)	1.393
Diabetes mellitus	10,574 (13.39%)	1985 (50.27%)	0.862
Ischemic heart disease	8239 (10.43%)	1180 (29.88%)	0.500
Hyperlipidemia	10,546 (13.35%)	1017 (25.75%)	0.317
Congestive heart failure	2650 (3.36%)	1282 (32.46%)	0.821
Cerebrovascular disease	5580 (7.07%)	728 (18.44%)	0.346
Dementia	915 (1.16%)	90 (2.28%)	0.086
Uveitis	116 (0.15%)	12 (0.30%)	0.033
Retinal vessel occlusion	94 (0.12%)	29 (0.73%)	0.095
NPDR	440 (0.56%)	241 (6.10%)	0.313
PDR Frequency of visits to ophthalmologists after index date 0 1 ≥2	153 (0.19%) 57,165 (72.38%) 8938 (11.32%) 12,877 (16.30%)	375 (9.50%) 2632 (66.65%) 433 (10.96) 884 (22.39%)	0.444 0.128

* The length of hospital stay and comorbidities were identified within one year before the index date. ASD, absolute standardized difference; NPDR, non-proliferative diabetic retinopathy; PDR, proliferative diabetic retinopathy.

**Table 2:** Incidence of glaucoma in the non-dialysis and dialysis groups.

	Non-Dialysis *n* = 78,980	Dialysis *n* = 3949
Follow-up person months	5,737,499	196,905
New glaucoma cases	2877	161
Incidence rate * (95% CI)	5.01 (4.83–5.2)	8.18 (7.01–9.54)
Crude relative risk (95% CI)	Reference	1.618 (1.38–1.897)

* Incidence rate, per 10,000 person months. CI, confidence interval.

**Table 3:** Multiple Cox proportional hazards regressions for the estimation of adjusted hazard ratios for glaucoma.

Variable	aHR (95% CI)
Dialysis (ref: Control)	1.270 (1.035–1.560)
Sex (ref: Female)	
Male	0.898 (0.836–0.965)
Age (ref: 40–59)	
20–39	0.445 (0.353–0.561)
60–79	2.069 (1.902–2.251)
80–100	1.399 (1.185–1.652)
Low income	0.968 (0.592–1.582)
Co-morbidity *	
Hypertension	1.133 (1.041–1.233)
Diabetes mellitus	1.524 (1.383–1.678)
Ischemic heart diseases	1.151 (1.035–1.279)
Hyperlipidemia	1.155 (1.046–1.276)
Uveitis	3.555 (2.203–5.737)
Retinal vessel occlusion	2.330 (1.315–4.128)

* Co-morbidity was identified within one year before index date. aHR, adjusted hazard ratio; CI, confidence interval.

**Table 4:** Subevent analysis in age- and sex-matched population.

	Incidence Rate * (95% CI)		
Sub-Event	Control	Dialysis	aHR + (95% CI)
OAG	0.94 (0.86–1.02)	1.53 (1.08–2.17)	1.008 (0.637–1.595)
NTG	0.18 (0.15–0.22)	0.29 (0.13–0.66)	0.901 (0.327–2.483)
ACG	1.52 (1.43–1.63)	2.28 (1.71–3.04)	1.550 (1.074–2.239)
Trabeculectomy	0.11 (0.08–0.14)	0.44 (0.23–0.85)	3.666 (1.366–9.840)

* Per 10000 person years; **+** adjusted for demographic variables, length of hospital stay, and comorbidities at baseline. ACG, angle-closure glaucoma; aHR, adjusted hazard ratio; CI, confidence interval; NTG, normal-tension glaucoma; OAG, open-angle glaucoma.
